# Laparoscopic radical nephroureterectomy in the oblique supine lithotomy integrative position for rare renal malignant perivascular epithelioid cell tumor with renal vein cancerous thrombosis: A case report and literature review

**DOI:** 10.1097/MD.0000000000030653

**Published:** 2022-09-23

**Authors:** Canbin Lin, Shulin Liang, Yongxing Wang, Aidi Liang, Weiting Qin, Jiapeng Huang, Hao Meng, Hong Liu, Ming Chen, Lei Meng

**Affiliations:** a Department of Urology, The First Affiliated Hospital of Guangzhou University of Chinese Medicine, Guangzhou, Guangdong, PR China; b The First Clinical Medical College, Guangzhou University of Chinese Medicine, Guangzhou, Guangdong, PR China; c Department of Traditional Chinese Medicine, Jinan University, Guangzhou, Guangdong, PR China.

**Keywords:** laparoscopic radical nephroureterectomy, malignant, oblique supine lithotomy position, perivascular epithelioid cell tumor, rare

## Abstract

Perivascular epithelioid cell tumor (PEComa) is a mesenchymal tumor that arises from perivascular epithelioid cells and can differentiate into melanocytes and smooth muscle cells. Malignant renal perivascular epithelioid cell tumor is extremely rare. Due to the lack of specific clinical manifestations and imaging features, diagnosing PEComa depends on postoperative pathology and immunohistochemistry. Surgery is the primary treatment for malignant PEComa because the efficacy of radiotherapy and chemotherapy is uncertain. There is still a lack of unified diagnostic criteria and treatment guidelines for renal malignant PEComa, especially with vascular invasion. Hence, the treatment experience depends on a small number of cases reported worldwide.

**Patient concerns:**

A 68-year-old woman was admitted to our hospital due to intermittent hematuria for over 8 months. The color Doppler ultrasound and computed tomography scan revealed a mass in the lower middle part of the left kidney.

**Diagnosis:**

Rare renal malignant perivascular epithelioid cell tumor with renal vein cancerous thrombosis.

**Interventions:**

A laparoscopic radical left nephroureterectomy in the oblique supine lithotomy position was performed.

**Outcomes:**

The operation process went smoothly, and no pulmonary embolism occurred after the operation. The final pathological diagnosis was a renal malignant perivascular epithelioid cell tumor. After a 12-month follow-up, no recurrence or metastasis was found.

**Lessons:**

Renal malignant PEComa is an extremely rare mesenchymal tumor diagnosed mainly based on pathology. Surgery is currently the effective treatment for malignant PEComa. For the surgical treatment of malignant renal PEComa with vascular invasion, laparoscopic radical nephroureterectomy in the oblique supine lithotomy integrative position has many benefits, as exemplified by our current case.

## 1. Introduction

Perivascular epithelioid cell tumor (PEComa) is a rare mesenchymal tumor that is more prevalent in women.^[1]^ According to the 2020 World Health Organization Classification of soft tissue tumors, PEComa includes perivascular epithelioid cell tumor-not otherwise specified, angiomyolipoma, and lymphangiomyomatosis. The differential diagnosis includes tumors with clear cell morphology and other spindle or epithelioid mesenchymal tumors that can be distinguished by clinical and morphological features and immunophenotype.^[2]^

Since no specific imaging features for diagnosing PEComa are currently available, it is difficult to make a definitive diagnosis without histological examination.^[3]^ PEComa has distinct histological features such as epithelioid cells with clear granular cytoplasm, eosinophilic, round to oval, and inconspicuous nuclei. Myogenic and melanocyte markers can also be detected by immunohistochemistry because epithelioid cells can differentiate into smooth muscle spindle cells or melanocytes.^[4]^

Renal PEComa mainly includes angiomyolipoma, which is relatively common.^[5]^ However, malignant renal perivascular epithelioid cell tumor is extremely rare,^[6–17]^ and only a few cases have been reported worldwide. Surgery is the primary treatment for malignant PEComa because the efficacy of chemoradiotherapy is uncertain. Moreover, there is still a lack of unified diagnostic criteria and treatment guidelines for renal malignant PEComa, especially for malignant PEComa with vascular invasion. Therefore, the treatment experience of renal malignant PEComa depends on a small number of cases reported worldwide.

Herein, we reported a case of renal malignant PEcoma with renal vein cancerous thrombosis in a woman who underwent laparoscopic radical nephroureterectomy in the oblique supine lithotomy integrative position and achieved good treatment results. It is worth mentioning that this operating method for renal malignant PEcoma with renal vein tumor thrombus was first established by our team.

## 2. Case presentation

A 68-year-old woman was presented with intermittent gross hematuria lasting >8 months and worsening for 1 month, accompanied by dizziness, palpitation, pallor, nausea, and vomiting. She was treated at a local hospital, and a blood transfusion was performed to correct anemia. The computed tomography (CT) revealed a left renal mass, and she came to our hospital for further treatment. In 2014, she underwent a right hip replacement at a local hospital. The physical examination did not reveal a lumboabdominal mass.

After admission to our hospital, the blood routine tests showed abnormal hemoglobin levels (82 g/L; normal range: 130–175 g/L). The urine routine tests presented a significant elevated red blood cell count (16,576 cells/µL; normal range: 0–7 cells/µL) and white blood cell count (256 cells/µL; normal range: 0–12 cells/µL). Next, we performed a color ultrasound and CT to evaluate the mass better.

**Figure 1.**
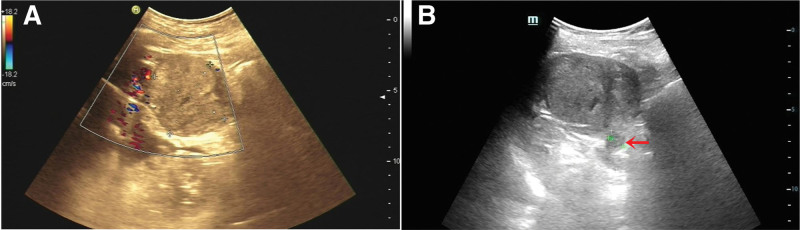
(A) Color Doppler ultrasound indicated a solid mass with blood flow in the middle part of the left kidney. (B) A hypoechoic mass in renal vein was indicated by the arrow.

**Figure 2.**
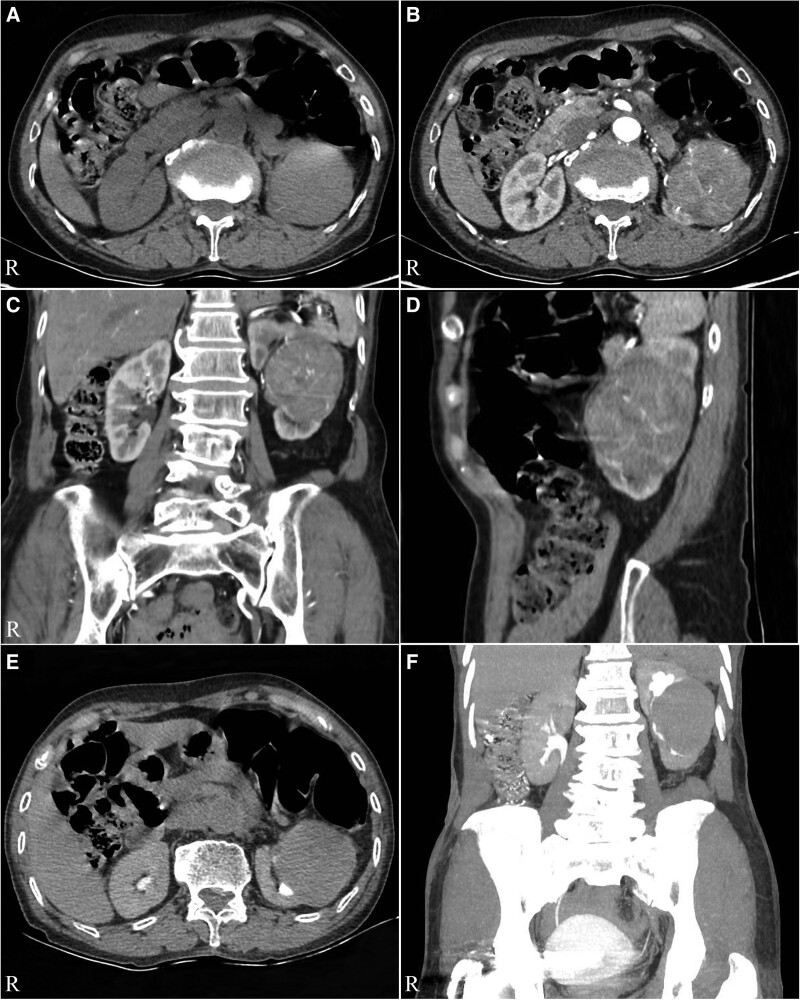
(A) CT indicated a mass in the lower middle part of the left kidney, about 72 mm × 62 mm in size. (B) CT with enhancement showed that the tumor was strengthened. (C) Coronal plane with enhancement. (D) Sagittal plane with enhancement. (E) CTU showed renal pelvis compression and mild hydronephrosis in the upper calyx. (F) Coronal plane of CTU. CT = computed tomography, CTU = computed tomography urography

**Figure 3.**
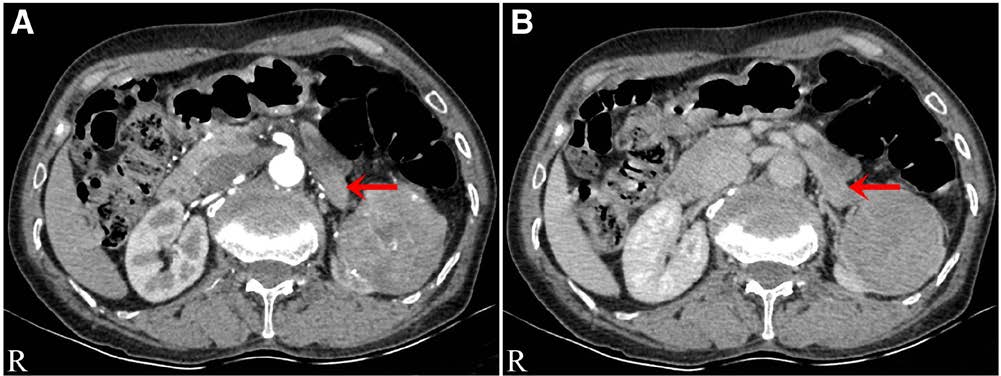
(A) The enhanced soft tissue shadow was found in the left renal vein in the arterial phase of the enhanced CT scan, as indicated by the arrow. (B) A filling defect was detected in the delayed CT scan, as indicated by arrows. CT = computed tomography

The Color Doppler ultrasound indicated a solid mass with blood flow in the middle part of the left kidney (Fig. 1A) and a hypoechoic mass in renal vein (Fig. 1B). The CT showed a 72 mm × 62 mm tumor in the middle and lower part of the left kidney protruding into the renal sinus (Fig. 2A). Uneven enhancement of the mass and striated vessels were detected on contrast-enhanced scans (Fig. 2B). The coronal and sagittal CT scan are shown in Figure 2C and D respectively. Moreover, the computed tomography urography showed renal pelvis compression and mild hydronephrosis in the upper calyx (Fig. 2E and F). In addition, enhanced soft tissue shadow was found in the left renal vein in the arterial phase of the enhanced CT scan (Fig. 3A), and a filling defect was detected in the delayed scan (Fig. 3B). No abnormalities were found in the proximal segment and inferior vena cava. The radionuclide renal dynamic imaging showed that the left glomerular filtration rate was 15.78 mL/min, and the right glomerular filtration rate was 29.52 mL/min (normal range: 80–120 mL/min).

In conclusion, the diagnosis was a renal malignant tumor invading the collecting system with renal vein tumor thrombus. The traditional surgical method applied in this situation is transurethral plasma sleeve resection of the ureteral wall on the affected side of the bladder in the lithotomy position. Then, the whole length of the affected kidney and ureter is removed in the lateral or oblique position. However, for this case, the traditional operation has some shortcomings. When the ureter has not been blocked, urine still flows out after the end of the ureter is free, and the urine containing cancer cells may extravasate outside the bladder incision, resulting in possible implantation and metastasis. Bladder incision can only continue to drain the bladder through the indwelling catheter to keep the bladder empty and heal by itself. During the operation, the lithotomy position needs to be changed to lateral or oblique decubitus position, which is laborious for the medical staff and increases the anesthesia and operation interruption times.

**Figure 4.**
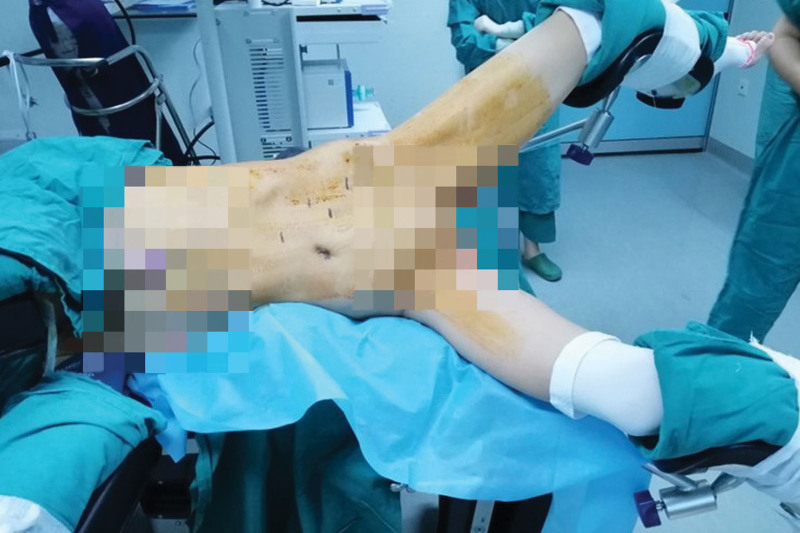
The oblique supine lithotomy integrative position.

Therefore, after a detailed plan formulation and careful preoperative preparation, such as transfusion therapy to correct anemia, the patient underwent laparoscopic radical nephroureterectomy in an integrative oblique supine lithotomy position (Fig. 4). The operation went smoothly and lasted about 495 min, and the intraoperative blood loss was about 300 mL.

**Figure 5.**
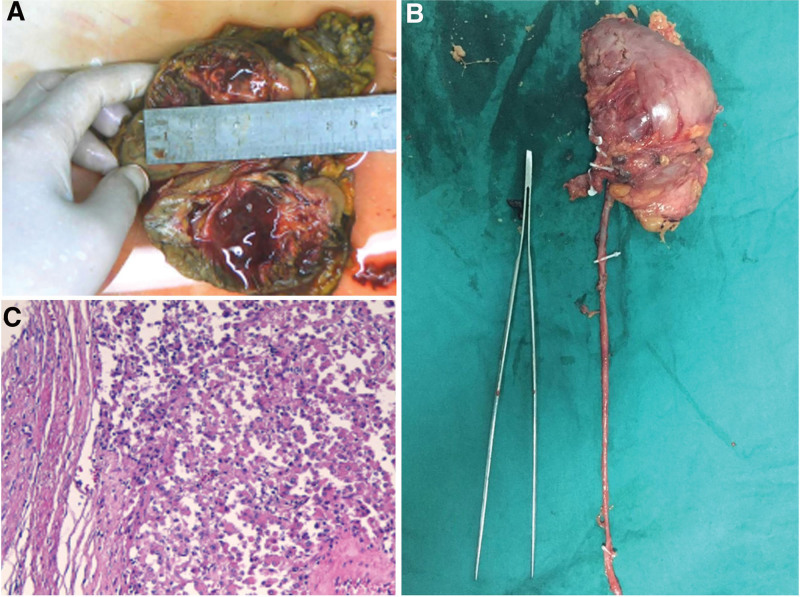
(A) The tumor specimen. (B) The resected specimen. (C) Pathological examination of the mass (hematoxylin and eosin staining; magnification, 200×).

The resected specimen shown in Fig. 5A and B. According to the pathological examination (Fig. 5C), the histological features were as follows: tumor cells were diffusely distributed around the blood vessels with oval and short spindle shapes; epithelioid cells varied in size, abundant cytoplasm, and eosinophilic; giant tumor cells were also observed; mitosis was rare with extensive degeneration and necrosis; there was a tumor thrombus in the vein (hematoxylin and eosin staining; magnification, 200×). The immunohistochemistry results were: CK (−), CK7 (−), CK8/18 (−), EMA (−), INI-1 (−), Desmin (−), Vimentin (+++), CD10 (±), HMB45 part (+), SMA part (+), and Ki-67 3% (+). Thus, it was finally diagnosed as renal malignant PEComa.

Finally, the patient recovered well without complications, and the hospital stay was 23 days. The patient did not experience discomfort during the 12-month follow-up and provided informed consent to open the case.

## 3. Discussion

In 2002, the World Health Organization defined perivascular epithelioid cell tumor (PEComa) as the mesenchymal tumor composed of perivascular epithelioid cells in histology and immunohistochemistry. PEComa mainly consists of clear eosinophilic epithelioid or spindle cells that grow in nests and bundles.^[1,2]^ Therefore, immunohistochemistry is crucial in the differential diagnosis between malignant PEcoma and renal cell carcinoma, renal sarcoma, or renal melanoma because it is easily confused with these malignancies on histologic evaluations. In immunohistochemistry, the coexpression of melanocyte and smooth muscle markers is the hallmark of PEComa.^[4,18]^

In the past, PEComa was often considered a benign tumor. However, it has been internationally reported that some PEcoma patients suffer from local recurrence and distant metastasis. The diagnosis of malignant PEComa should be considered when intratumoral bleeding and large areas of necrosis appear. Based on histological features such as lesion size, growth pattern, nuclear grade, mitotic activity, necrosis, and vascular invasion, PEComa can be classified into 3 types: benign, uncertain malignant potential, and malignant. Currently, the most useful features for predicting malignancy are tumor size >5 cm, invasive growth pattern, high nuclear grade and cellular structure, mitotic rate >1/50 (HPF), necrosis, and vascular invasion. In the presence of morphological features of malignancy, such as pleomorphism, necrosis, and mitotic activity, the term “malignant PEComa” is justified. The postoperative pathological findings suggested extensive degeneration and necrosis in our current case. Therefore, the final diagnosis was renal malignant PEComa.^[19,20]^

Malignant renal perivascular epithelioid cell tumor is extremely rare, and there is still a lack of unified diagnostic criteria and treatment guidelines for renal malignant PEComa, especially for malignancies with vascular invasion. Only a few cases have been reported worldwide and can be referred to in diagnosing and treating this disease.^[6–17]^ Characteristics of the 12 cases of renal malignant PEcoma from PubMed are shown in Table 1. According to relevant literature reports, the treatment strategy mainly depends on the tumor status, overall health status, and symptoms. Nevertheless, the optimal treatment is still controversial. A review of the current literature indicated that surgical resection with an adequate surgical margin is considered to provide the best outcome, and adjuvant therapy is usually not considered after surgical resection because the efficacy of chemoradiotherapy is uncertain.^[21]^

**Table 1:** Characteristics of the 12 cases of renal malignant PEcoma from PubMed.

Characteristics	Total	No. of patients (%)
Age (yr)		
≤40	6	50
>40	6	50
Gender		
male	5	41.67
female	7	58.33
Tumor size (cm)		
≤5.0	2	16.7
>5.0	10	83.3
Operation mode		
Radical nephrectomy	8	66.7
Partial nephrectomy	2	16.7
Refuse treatment	2	16.7
Primary/secondary		
Primary	8	66.7
Secondary	4	33.3
Tumor thrombus	2	16.7

PEComa = perivascular epithelioid cell tumor.

For the surgical treatment of renal cancer invading the collecting system, renal pelvis cancer, and ureteral cancer, the traditional standard surgical procedure is: the lithotomy position is performed with plasma cuff resection of the ureteral wall segment on the affected side of the bladder through the urethra, then changed to the lateral or oblique position for full-length resection of the affected kidney and ureter. However, the disadvantages of this procedure are as follows: when the ureter has not been blocked, urine still flows out after the end of the ureter is free, and the urine containing cancer cells may extravasate outside the bladder incision, resulting in possible implantation and metastasis; bladder incision can only continue to drain the bladder through the indwelling catheter to keep the bladder empty and heal by itself; during the operation, the lithotomy position needs to be changed to lateral or oblique decubitus position, which is laborious for the medical staff and increases the anesthesia and operation interruption times.

Herein, the malignant PEComa complicated with renal vein tumor thrombus needed to be blocked as soon as possible during surgery to prevent the risk of pulmonary embolism and even sudden death caused by the loss of tumor thrombus.^[22]^ Additionally, the ureter was blocked as early as possible to avoid the risk of urine leaking out of the bladder and metastasis.

Our team established for the first time the laparoscopic radical nephroureterectomy in an oblique supine lithotomy integrative position for the rare renal malignant PEComa with renal vein cancerous thrombosis. Through this case, we found that the advantages of this operation are: since the operation was performed in an oblique supine lithotomy position, there was no need to change the surgical position during the operation, saving the anesthesia operation time; blocking the renal vein close to the inferior vena cava during operation can completely remove tumor thrombus and prevent shedding; during the operation, the ureter was blocked first, then the sleeve resection of the ureter wall segment was performed to avoid the risk of urine extravasation around the bladder and tumor implantation and metastasis; after the full-length nephrectomy and ureter, the bladder incision was closed with absorbable suture to accelerate the healing of the bladder incision, reduce the postoperative bladder urine leakage, and reduce the incidence of pelvic or even abdominal infection.

In conclusion, renal malignant PEcoma is very rare, and its diagnosis and treatment pose great challenges. Currently, diagnosing renal malignant PEcoma mainly depends on pathological examination, especially immunohistochemistry. Surgery is an effective method for malignant renal PEComa, but there are great risks and difficulties in treating tumors with renal vein tumor thrombus. Therefore, the surgery plan is very important. Due to the risk of thrombus shedding and tumor implantation caused by traditional surgical methods, we proposed a new surgical method for renal malignant PEcoma with renal vein tumor thrombus: laparoscopic radical nephroureterectomy in oblique supine lithotomy integrative position. This new surgical approach may help surgeons remove tumors more thoroughly while avoiding thrombus shedding and minimizing the risk of tumor implantation. The success of this surgical method might also provide urologists with a new operation option.

## Author contributions

**Data curation:** Aidi Liang, Yongxing Wang.

**Investigation:** Aidi Liang, Hao Meng, Hong Liu, Jiapeng Huang, Weiting Qin, Yongxing Wang.

**Methodology:** Canbin Lin, Shulin Liang.

**Project administration:** Canbin Lin, Shulin Liang.

**Supervision:** Lei Meng, Ming Chen.

**Validation:** Hao Meng, Hong Liu.

**Writing – original draft:** Canbin Lin, Shulin Liang.

**Writing – review & editing:** Lei Meng, Ming Chen.
